# 3,5-Dinitro-*N*-(4-nitro­phen­yl)benzamide

**DOI:** 10.1107/S1600536810045915

**Published:** 2010-11-13

**Authors:** Yuehong Ren, Yu Zuo, Yonggang Xiang, Ruitao Zhu

**Affiliations:** a Department of Chemistry, Taiyuan Normal University, Taiyuan 030031, People’s Republic of China

## Abstract

In the title mol­ecule, C_13_H_8_N_4_O_7_, the amide fragment has an *anti* configuration. The mean planes of the two benzene rings form a dihedral angle of 7.78 (4)°. The mean planes of the three nitro groups are twisted by 6.82 (3), 5.01 (4) and 18.94 (7)° with respect to the benzene rings to which they are attached. In the crystal, mol­ecules are linked by weak inter­molecular N—H⋯O hydrogen bonds into chains along [100].

## Related literature

For background to the biological activity of *N*-substituted benzamides and their use in synthesis, see: Saeed *et al.* (2010 ▶). For related structures, see: Raza *et al.* (2010 ▶); Gowda *et al.* (2003 ▶). For standard bond-length data, see: Allen *et al.* (1987 ▶).
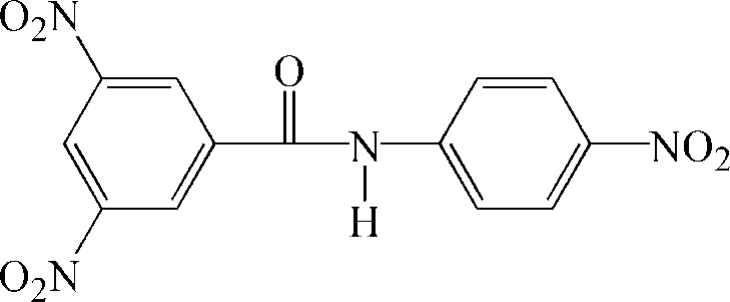


## Experimental

### 

#### Crystal data

C_13_H_8_N_4_O_7_
*M* *_r_* = 332.23
Monoclinic, 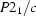

*a* = 7.8999 (9) Å
*b* = 8.019 (1) Å
*c* = 21.111 (2) Å
β = 94.285 (1)°
*V* = 1333.7 (3) Å^3^
*Z* = 4
Mo *K*α radiation
μ = 0.14 mm^−1^
*T* = 298 K
0.48 × 0.38 × 0.15 mm

#### Data collection

Bruker SMART CCD diffractometer
Absorption correction: multi-scan (*SADABS*; Sheldrick, 1996 ▶) *T*_min_ = 0.937, *T*_max_ = 0.980
6462 measured reflections
2361 independent reflections
1419 reflections with *I* > 2σ(*I*)
*R*_int_ = 0.055

#### Refinement

*R*[*F* ^2^ > 2σ(*F* ^2^)] = 0.046
*wR*(*F* ^2^) = 0.127
*S* = 1.01
2361 reflections
217 parameters
H-atom parameters constrained
Δρ_max_ = 0.22 e Å^−3^
Δρ_min_ = −0.22 e Å^−3^

### 

Data collection: *SMART* (Bruker, 2007 ▶); cell refinement: *SAINT* (Bruker, 2007 ▶); data reduction: *SAINT*; program(s) used to solve structure: *SHELXS97* (Sheldrick, 2008 ▶); program(s) used to refine structure: *SHELXL97* (Sheldrick, 2008 ▶); molecular graphics: *SHELXTL* (Sheldrick, 2008 ▶) and *PLATON* (Spek, 2009 ▶); software used to prepare material for publication: *SHELXTL*.

## Supplementary Material

Crystal structure: contains datablocks I, global. DOI: 10.1107/S1600536810045915/lh5155sup1.cif

Structure factors: contains datablocks I. DOI: 10.1107/S1600536810045915/lh5155Isup2.hkl

Additional supplementary materials:  crystallographic information; 3D view; checkCIF report

## Figures and Tables

**Table 1:** Hydrogen-bond geometry (Å, °)

*D*—H⋯*A*	*D*—H	H⋯*A*	*D*⋯*A*	*D*—H⋯*A*
N1—H1⋯O2^i^	0.86	2.52	3.280 (3)	147

Symmetry code: (i) 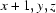.

**Fig. 1.**
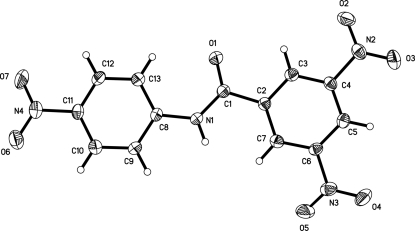
The molecular structure of (I), displacement ellipsoids are drawn at the 30% probability level.

**Fig. 2.**
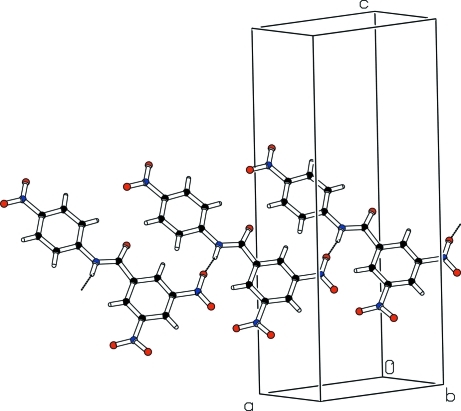
Part of the crystal structure of (I) with hydrogen bonds drawn as dashed lines.

## supplementary crystallographic information

### Comment

N-substituted benzamides have numerous pharmaceutical and synthetic application (Saeed *et al.* 2010). In this paper, we report the structure of the title compound (I). The molecular structure of (I) is shown in Fig. 1. The bond lengths are within normal ranges (Allen *et al.* 1987). The amide N—H and C═O bonds in the molecule comprising the crystallographic asymmetric unit are *trans* to each other and similar to those observed in 2-Hydroxy-*N*-(3-nitrophenyl)benzamide (Raza *et al.* 2010) and 2-chloro-*N*-(phenyl)-benzamide (NP2CBA) (Gowda *et al.*, 2003). The mean planes of the two benzene rings form a dihedral angle of 7.78 (4)°. The mean planes of the three nitro groups are twisted by 6.82 (3)°, 5.01 (4)° and 18.94 (7)° with respect to the benzene rings to which they are attached. In the crystal structure, molecules are linked by weak intermolecular N-H···O hydrogen bonds in chains along [100] (see Fig. 2).

### Experimental

3,5-Dinitrobenzoyl chloride (1.15 g, 5 mmol) dissolved in tetrahydrofuran (10 ml) was added to 4-nitroaniline (0.69 g, 5 mmol) dissolved in tetrahydrofuran (5 ml), the reaction mixture was refluxed for 2 h, then cooled to ambient temperature and filtered to remove the tetrahydrofuran. The precipitate was dissolved in methanol/tetrahydrofuran/ethyl acetate (1:1:1) and the solution was allowed to stand for a few days at ambient temperature, after which time colorless plates of the title compound suitable for X-ray diffraction were obtaind.

### Refinement

H atoms were placed in idealized positions and allowed to ride on theirrespective parent atoms, with C—H = 0.93Å, N-H = 0.86Å and with *U*_iso_(H) = 1.2*U*_eq_(C,N).

### Crystal data

**Table:**

C_13_H_8_N_4_O_7_	*F*(000) = 680
*M**_r_* = 332.23	*D*_x_ = 1.655 Mg m^−^^3^
Monoclinic, *P*2_1_/*c*	Mo *K*α radiation, λ = 0.71073 Å
Hall symbol: -P 2ybc	Cell parameters from 1593 reflections
*a* = 7.8999 (9) Å	θ = 2.6–26.4°
*b* = 8.019 (1) Å	µ = 0.14 mm^−^^1^
*c* = 21.111 (2) Å	*T* = 298 K
β = 94.285 (1)°	Plate, colorless
*V* = 1333.7 (3) Å^3^	0.48 × 0.38 × 0.15 mm
*Z* = 4	

### Data collection

**Table:**

Bruker SMART CCD diffractometer	2361 independent reflections
Radiation source: fine-focus sealed tube	1419 reflections with *I* > 2σ(*I*)
graphite	*R*_int_ = 0.055
φ and ω scans	θ_max_ = 25.0°, θ_min_ = 2.6°
Absorption correction: multi-scan (*SADABS*; Sheldrick, 1996)	*h* = −9→9
*T*_min_ = 0.937, *T*_max_ = 0.980	*k* = −9→9
6462 measured reflections	*l* = −25→17

### Refinement

**Table:**

Refinement on *F*^2^	Primary atom site location: structure-invariant direct methods
Least-squares matrix: full	Secondary atom site location: difference Fourier map
*R*[*F*^2^ > 2σ(*F*^2^)] = 0.046	Hydrogen site location: inferred from neighbouring sites
*wR*(*F*^2^) = 0.127	H-atom parameters constrained
*S* = 1.01	*w* = 1/[σ^2^(*F*_o_^2^) + (0.0524*P*)^2^ + 0.2955*P*] where *P* = (*F*_o_^2^ + 2*F*_c_^2^)/3
2361 reflections	(Δ/σ)_max_ < 0.001
217 parameters	Δρ_max_ = 0.22 e Å^−^^3^
0 restraints	Δρ_min_ = −0.22 e Å^−^^3^

### Special details

**Table:**

Geometry. All e.s.d.'s (except the e.s.d. in the dihedral angle between two l.s. planes) are estimated using the full covariance matrix. The cell e.s.d.'s are taken into account individually in the estimation of e.s.d.'s in distances, angles and torsion angles; correlations between e.s.d.'s in cell parameters are only used when they are defined by crystal symmetry. An approximate (isotropic) treatment of cell e.s.d.'s is used for estimating e.s.d.'s involving l.s. planes.
Refinement. Refinement of *F*^2^ against ALL reflections. The weighted *R*-factor *wR* and goodness of fit *S* are based on *F*^2^, conventional *R*-factors *R* are based on *F*, with *F* set to zero for negative *F*^2^. The threshold expression of *F*^2^ > σ(*F*^2^) is used only for calculating *R*-factors(gt) *etc*. and is not relevant to the choice of reflections for refinement. *R*-factors based on *F*^2^ are statistically about twice as large as those based on *F*, and *R*- factors based on ALL data will be even larger.

### Fractional atomic coordinates and isotropic or equivalent isotropic displacement parameters (Å^2^)

**Table:**

	*x*	*y*	*z*	*U*_iso_*/*U*_eq_	
N1	1.4744 (2)	0.3431 (3)	0.43550 (9)	0.0417 (6)	
H1	1.5292	0.3850	0.4056	0.050*	
N2	0.8118 (3)	0.6627 (3)	0.33003 (12)	0.0470 (6)	
N3	1.3396 (3)	0.6676 (4)	0.22261 (10)	0.0503 (7)	
N4	1.8925 (3)	0.0058 (3)	0.61994 (11)	0.0498 (7)	
O1	1.2103 (2)	0.3154 (3)	0.46823 (9)	0.0623 (7)	
O2	0.7337 (2)	0.6051 (3)	0.37295 (10)	0.0605 (6)	
O3	0.7509 (3)	0.7558 (3)	0.28922 (11)	0.0735 (8)	
O4	1.2863 (3)	0.7866 (3)	0.19062 (10)	0.0702 (7)	
O5	1.4673 (3)	0.5907 (3)	0.21316 (9)	0.0737 (8)	
O6	2.0458 (3)	0.0098 (3)	0.61510 (10)	0.0680 (7)	
O7	1.8282 (3)	−0.0657 (4)	0.66238 (11)	0.0845 (9)	
C1	1.3044 (3)	0.3678 (3)	0.43039 (12)	0.0381 (7)	
C2	1.2337 (3)	0.4698 (3)	0.37448 (11)	0.0340 (6)	
C3	1.0657 (3)	0.5183 (3)	0.37588 (12)	0.0376 (7)	
H3	1.0034	0.4865	0.4096	0.045*	
C4	0.9913 (3)	0.6136 (3)	0.32735 (12)	0.0362 (6)	
C5	1.0782 (3)	0.6669 (3)	0.27726 (11)	0.0385 (7)	
H5	1.0272	0.7341	0.2454	0.046*	
C6	1.2440 (3)	0.6163 (3)	0.27634 (11)	0.0359 (6)	
C7	1.3229 (3)	0.5177 (3)	0.32335 (11)	0.0374 (7)	
H7	1.4347	0.4837	0.3207	0.045*	
C8	1.5729 (3)	0.2579 (3)	0.48343 (11)	0.0343 (6)	
C9	1.7451 (3)	0.2441 (4)	0.47600 (13)	0.0434 (7)	
H9	1.7894	0.2898	0.4403	0.052*	
C10	1.8513 (3)	0.1638 (4)	0.52054 (12)	0.0441 (7)	
H10	1.9671	0.1561	0.5157	0.053*	
C11	1.7825 (3)	0.0952 (3)	0.57246 (12)	0.0374 (7)	
C12	1.6121 (3)	0.1071 (4)	0.58118 (12)	0.0419 (7)	
H12	1.5688	0.0596	0.6168	0.050*	
C13	1.5060 (3)	0.1896 (4)	0.53691 (11)	0.0413 (7)	
H13	1.3908	0.1997	0.5426	0.050*	

### Atomic displacement parameters (Å^2^)

**Table:**

	*U*^11^	*U*^22^	*U*^33^	*U*^12^	*U*^13^	*U*^23^
N1	0.0368 (12)	0.0543 (17)	0.0345 (12)	0.0023 (11)	0.0061 (9)	0.0137 (11)
N2	0.0389 (13)	0.0526 (18)	0.0483 (15)	0.0044 (12)	−0.0050 (11)	−0.0092 (13)
N3	0.0473 (15)	0.071 (2)	0.0325 (14)	−0.0119 (14)	0.0026 (11)	−0.0005 (14)
N4	0.0538 (16)	0.0493 (18)	0.0445 (15)	0.0019 (13)	−0.0091 (12)	0.0000 (12)
O1	0.0413 (11)	0.0895 (18)	0.0571 (13)	0.0032 (11)	0.0099 (10)	0.0337 (13)
O2	0.0436 (11)	0.0765 (17)	0.0632 (14)	0.0030 (11)	0.0155 (10)	−0.0032 (12)
O3	0.0575 (14)	0.091 (2)	0.0701 (16)	0.0252 (13)	−0.0085 (11)	0.0178 (14)
O4	0.0778 (16)	0.0818 (19)	0.0513 (14)	−0.0048 (14)	0.0066 (11)	0.0273 (13)
O5	0.0576 (14)	0.112 (2)	0.0538 (14)	0.0055 (14)	0.0224 (11)	0.0070 (13)
O6	0.0534 (14)	0.0822 (19)	0.0665 (15)	0.0147 (12)	−0.0085 (11)	0.0043 (12)
O7	0.0761 (16)	0.112 (2)	0.0634 (16)	−0.0021 (15)	−0.0101 (12)	0.0486 (15)
C1	0.0375 (15)	0.0405 (18)	0.0362 (15)	−0.0014 (13)	0.0024 (12)	0.0030 (13)
C2	0.0344 (14)	0.0354 (17)	0.0318 (14)	−0.0021 (12)	0.0007 (11)	−0.0007 (12)
C3	0.0366 (14)	0.0404 (18)	0.0359 (15)	−0.0042 (13)	0.0028 (11)	−0.0008 (13)
C4	0.0303 (13)	0.0412 (18)	0.0365 (15)	0.0007 (12)	−0.0025 (11)	−0.0056 (13)
C5	0.0418 (15)	0.0427 (18)	0.0298 (15)	−0.0015 (13)	−0.0060 (11)	−0.0019 (13)
C6	0.0390 (15)	0.0422 (18)	0.0267 (14)	−0.0065 (13)	0.0029 (11)	−0.0010 (12)
C7	0.0332 (13)	0.0425 (18)	0.0360 (15)	−0.0037 (12)	0.0006 (11)	−0.0061 (13)
C8	0.0388 (15)	0.0339 (17)	0.0298 (14)	0.0015 (12)	0.0004 (11)	0.0033 (12)
C9	0.0429 (16)	0.051 (2)	0.0373 (15)	0.0016 (13)	0.0080 (12)	0.0113 (13)
C10	0.0410 (15)	0.049 (2)	0.0419 (16)	0.0017 (14)	0.0003 (12)	0.0015 (14)
C11	0.0434 (15)	0.0331 (17)	0.0342 (15)	0.0018 (12)	−0.0070 (12)	−0.0017 (12)
C12	0.0497 (17)	0.0460 (19)	0.0300 (15)	−0.0038 (14)	0.0029 (12)	0.0044 (13)
C13	0.0401 (15)	0.0472 (19)	0.0366 (15)	0.0004 (13)	0.0037 (12)	0.0018 (14)

### Geometric parameters (Å, °)

**Table:**

N1—C1	1.354 (3)	C3—H3	0.9300
N1—C8	1.407 (3)	C4—C5	1.371 (3)
N1—H1	0.8600	C5—C6	1.373 (3)
N2—O3	1.212 (3)	C5—H5	0.9300
N2—O2	1.224 (3)	C6—C7	1.381 (3)
N2—C4	1.476 (3)	C7—H7	0.9300
N3—O5	1.212 (3)	C8—C9	1.385 (3)
N3—O4	1.225 (3)	C8—C13	1.394 (3)
N3—C6	1.467 (3)	C9—C10	1.373 (4)
N4—O7	1.207 (3)	C9—H9	0.9300
N4—O6	1.224 (3)	C10—C11	1.374 (3)
N4—C11	1.464 (3)	C10—H10	0.9300
O1—C1	1.206 (3)	C11—C12	1.376 (3)
C1—C2	1.509 (3)	C12—C13	1.377 (3)
C2—C3	1.386 (3)	C12—H12	0.9300
C2—C7	1.386 (3)	C13—H13	0.9300
C3—C4	1.375 (4)		
			
C1—N1—C8	128.3 (2)	C6—C5—H5	121.5
C1—N1—H1	115.8	C5—C6—C7	122.6 (2)
C8—N1—H1	115.8	C5—C6—N3	118.3 (2)
O3—N2—O2	124.3 (2)	C7—C6—N3	119.1 (2)
O3—N2—C4	117.9 (2)	C6—C7—C2	119.4 (2)
O2—N2—C4	117.8 (2)	C6—C7—H7	120.3
O5—N3—O4	124.2 (2)	C2—C7—H7	120.3
O5—N3—C6	117.9 (3)	C9—C8—C13	119.7 (2)
O4—N3—C6	118.0 (3)	C9—C8—N1	116.9 (2)
O7—N4—O6	123.2 (2)	C13—C8—N1	123.4 (2)
O7—N4—C11	118.7 (2)	C10—C9—C8	121.0 (2)
O6—N4—C11	118.1 (2)	C10—C9—H9	119.5
O1—C1—N1	123.6 (2)	C8—C9—H9	119.5
O1—C1—C2	119.8 (2)	C9—C10—C11	118.4 (2)
N1—C1—C2	116.6 (2)	C9—C10—H10	120.8
C3—C2—C7	118.8 (2)	C11—C10—H10	120.8
C3—C2—C1	115.8 (2)	C10—C11—C12	121.9 (2)
C7—C2—C1	125.4 (2)	C10—C11—N4	119.5 (2)
C4—C3—C2	119.8 (2)	C12—C11—N4	118.6 (2)
C4—C3—H3	120.1	C11—C12—C13	119.6 (2)
C2—C3—H3	120.1	C11—C12—H12	120.2
C5—C4—C3	122.5 (2)	C13—C12—H12	120.2
C5—C4—N2	119.0 (2)	C12—C13—C8	119.3 (2)
C3—C4—N2	118.6 (2)	C12—C13—H13	120.3
C4—C5—C6	116.9 (2)	C8—C13—H13	120.3
C4—C5—H5	121.5		
			
C8—N1—C1—O1	−0.7 (5)	O4—N3—C6—C7	−161.6 (3)
C8—N1—C1—C2	178.0 (2)	C5—C6—C7—C2	−1.4 (4)
O1—C1—C2—C3	9.6 (4)	N3—C6—C7—C2	179.6 (2)
N1—C1—C2—C3	−169.1 (2)	C3—C2—C7—C6	2.0 (4)
O1—C1—C2—C7	−170.7 (3)	C1—C2—C7—C6	−177.7 (2)
N1—C1—C2—C7	10.6 (4)	C1—N1—C8—C9	177.2 (3)
C7—C2—C3—C4	−0.6 (4)	C1—N1—C8—C13	−3.2 (4)
C1—C2—C3—C4	179.1 (2)	C13—C8—C9—C10	0.0 (4)
C2—C3—C4—C5	−1.4 (4)	N1—C8—C9—C10	179.6 (3)
C2—C3—C4—N2	179.7 (2)	C8—C9—C10—C11	0.9 (4)
O3—N2—C4—C5	−3.8 (4)	C9—C10—C11—C12	−1.0 (4)
O2—N2—C4—C5	175.4 (2)	C9—C10—C11—N4	178.7 (3)
O3—N2—C4—C3	175.2 (3)	O7—N4—C11—C10	−173.8 (3)
O2—N2—C4—C3	−5.6 (4)	O6—N4—C11—C10	7.4 (4)
C3—C4—C5—C6	1.9 (4)	O7—N4—C11—C12	5.8 (4)
N2—C4—C5—C6	−179.1 (2)	O6—N4—C11—C12	−173.0 (2)
C4—C5—C6—C7	−0.5 (4)	C10—C11—C12—C13	0.1 (4)
C4—C5—C6—N3	178.5 (2)	N4—C11—C12—C13	−179.6 (3)
O5—N3—C6—C5	−161.5 (3)	C11—C12—C13—C8	0.9 (4)
O4—N3—C6—C5	19.3 (4)	C9—C8—C13—C12	−0.9 (4)
O5—N3—C6—C7	17.5 (4)	N1—C8—C13—C12	179.5 (3)

### Hydrogen-bond geometry (Å, °)

**Table:**

*D*—H···*A*	*D*—H	H···*A*	*D*···*A*	*D*—H···*A*
N1—H1···O2^i^	0.86	2.52	3.280 (3)	147

Symmetry codes: (i) *x*+1, *y*, *z*.
